# A Case of Severe Polyhydramnios During Pregnancy Associated with Long-Term Use of Lithium

**DOI:** 10.1192/j.eurpsy.2024.1668

**Published:** 2024-08-27

**Authors:** A. Stoppa, E. Roda, A. S. Hernández, A. Roca

**Affiliations:** ^1^Perinatal Mental Health Unit; ^2^Department of Maternal-Fetal Medicine, Hospital Clinic of Barcelona, Barcelona, Spain

## Abstract

**Introduction:**

The perinatal period poses heightened vulnerability to bipolar affective episodes. Lithium serves as first line in the management of bipolar disorder, demonstrating efficacy in stabilizing mood episodes and preventing relapses. Therefore, it also a recommended treatment during the pregnancy period. However, its use presents potential risks for both the mother and the developing fetus. Given the prevalence of bipolar disorder in reproductive-age women, it is crucial to investigate the risks associated with lithium use during pregnancy, along with its subsequent obstetric and neonatal complications.

**Objectives:**

This report outlines a case of severe polyhydramnios in a 42-year-old primigravida patient, under long-term lithium and antipsychotic treatment. Additionally, a systematic search for similar case reports was conducted to provide an overview of the existing literature.

**Methods:**

The patient’s medical history and perinatal medical care are documented in this case report. A systematic literature search on MEDLINE (PubMed) was conducted using Boolean operators.

**Results:**

The patient was diagnosed with bipolar disorder type I and had a history of lithium treatment for over 20 years, supplemented later with antipsychotics. During her pregnancy, she experienced a polyuria-polydipsia syndrome and a severe polyhydramnios. She also suffered renal impairment. Together, it is indicative of a nephrogenic diabetes insipidus (NDI), likely induced by prolonged lithium treatment. As the pregnancy progressed, she experienced premature rupture of membranes at 34 weeks and 5 days. The newborn needed medical support and was admitted to the neonatal unit, without further complications.

Systematic research showed three published case reports describing nephrogenic diabetes insipidus (NDI) and polyhydramnios associated to lithium treatment.

**Conclusions:**

Chronic administration of lithium may contribute to the development of resistance to antidiuretic hormone (ADH), leading to polyuria-polydipsia syndrome and potentially severe obstetric complications. The co-administration of lithium and antipsychotics may exacerbate these effects. Further research is needed to elucidate their combined clinical impacts.

**Disclosure of Interest:**

None Declared

